# Psychosocial Support Needs and Utilization of Support Among Survivors of Cancer in Young Adulthood in Switzerland: A Report From the Adolescent and Young Adult (AYA) Psychosocial Health Study

**DOI:** 10.1002/pon.70399

**Published:** 2026-02-10

**Authors:** Céline Bolliger, Martina Ospelt, Marcel Blum, Oliver Gautschi, Luzius Mader, Walter Mingrone, Mohsen Mousavi, Beat Müller, Marcus Vetter, Katharina Roser

**Affiliations:** ^1^ Faculty of Health Sciences and Medicine University of Lucerne Lucerne Switzerland; ^2^ Cancer Registry of Eastern Switzerland St. Gallen Switzerland; ^3^ Department for Medical Oncology Cantonal Hospital of Lucerne Lucerne Switzerland; ^4^ Department for Medical Oncology University of Bern Berne Switzerland; ^5^ Cancer Registry Bern and Solothurn Bern Switzerland; ^6^ Department for Oncology Cantonal Hospital Olten Olten Switzerland; ^7^ Center for Oncology and Hematology University Hospital Basel Basel Switzerland

**Keywords:** adolescent and young adult cancer, cancer care, psychosocial needs, support needs, survivors

## Abstract

**Background:**

The psychosocial needs of survivors of cancer in young adulthood in Switzerland remain poorly understood. With this study, we aimed to (1) describe psychosocial support needs of young adult cancer survivors (YACS) during treatment and survivorship, (2) determine whether these needs change from treatment to survivorship, and (3) identify characteristics associated with unmet needs.

**Methods:**

We included survivors aged 21–39 years at diagnosis, diagnosed at least two years prior to our questionnaire survey. We used descriptive statistics to analyze distributions of psychosocial support needs during treatment and survivorship. McNemar's tests assessed changes in these needs over time, and logistic regressions identified determinants of unmet needs.

**Results:**

A total of 131 YACS (mean age at study = 37.5 years; 61% female) participated in our study. Most YACS reported that their needs were met in all support domains, both during treatment and survivorship. During treatment, unmet needs were highest for insurance support (*n* = 30; 23%), support for friends (*n* = 20; 15%) and family (*n* = 17; 13%). During survivorship, unmet needs were most pronounced for insurance support (*n* = 15; 12%), information on cancer and late effects (*n* = 10; 8%), and work‐related support (*n* = 9; 7%). Intrapersonal needs (e.g., information, psychological), remained stable, while unmet interpersonal (e.g., family, friends), and institutional/systemic (e.g., insurance, work) support needs declined during survivorship. Younger age and shorter time since diagnosis were associated with unmet intrapersonal needs during survivorship, while male sex predicted unmet interpersonal needs during treatment.

**Conclusion:**

Although most support needs are met, targeted efforts are needed to address the remaining unmet needs of Swiss YACS in order to ensure they receive adequate survivorship care.

**Abbreviations**
AYAs: Adolescent and young adults
CI: Confidence interval
CNS: Central nervous system
N: Numbers
OR: Odds ratio
Ref: Reference category
SCCSS: Swiss Childhood Cancer Survivor Study
YACS: Young adult cancer survivors

## 1 Background

Adolescents and young adults (AYAs, aged 15–39 years) diagnosed with cancer exhibit a distinct distribution of cancer types and face unique psychosocial challenges [1]. Cancer during this life phase may disrupt key developmental milestones related to education, work, relationships, and family planning with potential long‐term consequences for their quality of life and potentially creating support needs [2, 3, 4, 5].

Previous studies consistently report challenges related to educational and occupational trajectories, financial strain, access to tailored information, and long‐term planning among AYA cancer survivors [6, 7, 8, 9]. Furthermore, cancer can impact family and social relationships and raise concerns regarding fertility and future parenthood, issues that are particularly salient during young adulthood [7, 8, 10, 11, 12, 13].

In order to address these challenges effectively, it is essential to provide psychosocial support that is developmentally appropriate, flexible, and holistic across the cancer continuum for AYAs with cancer in need [14, 15]. A systematic review emphasizes the importance of adjusting information to the evolving needs of AYA cancer survivors throughout their cancer journey, highlighting the need for timely, proactive communication and stage‐specific support [16].

In Switzerland, the provision of psychosocial support for people affected by cancer is comparatively well‐established. Nationally, multiple organizations provide information and support to people with cancer. Eighteen national and regional cancer leagues provide counseling and support services free of charge [17]. Regarding services specific for AYAs with cancer, AYA Cancer Support CH, a Swiss association founded in 2021, offers AYA‐specific services, including bundled information, educational programs, peer support, and events for AYAs and their relatives [18]. Currently, AYA cancer care in Switzerland is delivered across both pediatric and adult oncology settings, with patients managed according to tumor type and institutional structures rather than within a dedicated or standardized nationwide AYA‐specific care framework. However, initial institutional initiatives aiming to improve coordination of AYA care are emerging [19]. In parallel, recent European initiatives have demonstrated growing momentum toward harmonised AYA cancer care through the development of minimum standards and implementation frameworks across diverse health care systems [20].

Despite the broad availability of psychosocial resources for cancer patients and survivors in general, it remains unclear to what extent existing services adequately address the evolving specific psychosocial needs of AYAs with cancer during treatment and survivorship. The assessment of unmet needs within a health care system that is well‐resourced may provide significant insights into the persistent challenges faced by AYAs with cancer and facilitate comparisons with countries where psychosocial support resources are more limited.

In this study, we aimed to (1) describe psychosocial support needs of survivors of young adult cancer (young adult cancer survivors; YACS) during treatment and survivorship, (2) determine whether these needs change from treatment to survivorship, and (3) identify characteristics associated with unmet needs.

## 2 Methods

### 2.1 Sample and Procedure

Study participants were recruited in collaboration with hospitals in the German‐speaking part of Switzerland. The identification of the clinics was facilitated with the support of regional Swiss cancer registries. Clinicians treating AYAs with cancer were invited to identify former patients (YACS) and send them an invitation to participate in the present study. Eligible survivors were aged 21–39 years at the time of diagnosis, had received treatment in Switzerland, and were German‐speaking. They were diagnosed between 2010–2019, and survived for ≥ 2 years after diagnosis. Survivors aged 15–20 years at the time of diagnosis are covered by the Swiss Childhood Cancer Survivor Study (SCCSS) [21].The present study therefore focused on YACS. From February 2023 to June 2024, YACS were sent a study package, which comprised an information letter, a consent form, a questionnaire, and a link to an online version of the questionnaire, in addition to a pre‐paid return envelope. Six weeks later, a reminder letter was sent to those who had not yet provided a response. For participants who had provided informed consent for the use of their cancer‐related data, additional information was obtained from the cancer registries. This was used to validate and complete their questionnaire responses. All procedures performed in this study were in accordance with the ethical standards of the responsible research committee and with the 1964 Helsinki Declaration and its later amendments or comparable ethical standards. The study was approved by the Ethics Committee Northwest and Central Switzerland (EKNZ 2022–01065, 12 October 2022).

### 2.2 Measurements

The questionnaire consisted of a total of 185 items and was developed using a combination of standardized and self‐developed questions regarding survivors' psychosocial health and needs, and their socio‐demographic and cancer‐related characteristics.

#### 2.2.1 Psychosocial Needs

To ascertain the psychosocial support needs of YACS during treatment and survivorship (at time of study) (Supporting Information S1: Table S1), the questionnaire employed 10 key subdomains of support: (1) information about cancer and its late effects, (2) psychological support, (3) educational assistance, (4) employment‐related support, (5) insurance guidance, (6) housing support, (7) partnership support, (8) support from family, (9) family planning assistance, and (10) support for friends. We categorized these subdomains into three overarching support domains: *Intrapersonal*: information about cancer and its late effects and psychological support, *Interpersonal:* partnership support, family support, family planning support, and support for friends, and *Institutional/systemic:* educational support, work‐related support, insurance support, and housing support.

For each subdomain, respondents selected one of the following response options: “I used it,” “It was available, I could have used it, but I did not,” “It was available, but I did not need it,” “It was not available, but I would have been interested,” and “I did not need it.” We categorized these response options into four subcategories for need: (a) *Used (available),* including “I used it”, (b) *Not used (available)*, including “It was available, I could have used it, but I did not” (c) *Not needed*, including “It was available, but I did not need it” and “I did not need it”; and (d) *Needed (not available),* including “It was not available, but I would have been interested”. These subcategories were summarized into two overarching need categories: *Unmet needs* (Needed (not available)) and *Met needs* (Used, Not used (available), and Not needed).

In open‐ended questions, we asked survivors if there were additional areas in which they wished they had received support during their cancer treatment and/or today, that is during cancer survivorship.

#### 2.2.2 Sociodemographic and Cancer‐Related Characteristics

The sociodemographic characteristics assessed in the questionnaire included the survivors' age at study (assessed continuously; categorized into 21–30, 31–39, ≥ 40 years), gender (female, male diverse), highest educational achievement (compulsory schooling, vocational training, upper secondary education, university degree), partnership status (yes, no), and migration background (yes, no; survivors were classified as having a migration background if they were not Swiss citizens, were not Swiss citizens since birth or were not born in Switzerland). For the analysis, the highest educational achievement was categorized as low (compulsory schooling, vocational training) or high (upper secondary education, university education), and employment status as employed (full‐time, part‐time) or other (homeworker, unemployed).

We further assessed information on diagnosis (classified according to the AYA cancer classification by Barr et al.(2020) [22]: leukemias and related disorders, lymphomas, CNS and other intracranial and intraspinal neoplasms, sarcomas, blood and lymphatic vessel tumors, nerve sheath tumors, gonadal and related tumors, malignant melanoma, carcinomas). For the analysis, we categorized the diagnosis information into carcinomas, gonadal tumors, lymphomas, and other tumors (leukemias, CNS neoplasms, sarcomas, nerve sheath tumors, and malignant melanoma). We assessed information on age at diagnosis (assessed continuously; categorized into 21–30, 31–39 years) and time since diagnosis (assessed continuously; categorized into 2–5, 6–9, 10–13 years). Treatment was assessed and hierarchically coded as surgery only, chemotherapy (may have had surgery), radiotherapy (may have had surgery and/or chemotherapy), and stem cell transplantation (may have had surgery and/or chemotherapy and/or radiotherapy). For the analysis, radiotherapy and stem cell transplantation were grouped together. We asked survivors whether they had experienced any late effects, cancer relapses, or second malignancies (yes, no). For *n* = 103 survivors, cancer‐related data from the corresponding cancer registry were available.

### 2.3 Statistical Analysis

We performed all statistical analyses using Stata 19.0 (StataCorp, TX). We used descriptive statistics to describe the study population. Where cancer‐related data from the questionnaire was missing (diagnosis: *n* = 2, year of diagnosis: *n* = 4, treatment: *n* = 3, relapse: *n* = 3, second cancer: *n* = 3), information was imputed using cancer registry data, where available. Additionally, we checked and validated the cancer‐related data from the questionnaire for the survivors for whom registry data was available, adding information from the registry where the questionnaire response was incomplete (treatment: *n* = 19; no other discrepancies). We used descriptive statistics and chi‐square tests to compare our YACS sample with the corresponding YACS population in Switzerland.

For aim 1, we used frequency distributions and percentages for each subcategory of need (*Used (available), Not used (available), Not needed, Needed (not available))* and for the overarching categories of need (*Unmet needs, Met needs*). Additionally, answers to open‐ended questions were coded according to their content by one researcher and checked by a second researcher, enabling the completion and clarification of information in the questionnaire.

For aim 2, changes in psychosocial support needs from treatment to survivorship were assessed using McNemar's tests across consecutive time points for the three support domains: *Intrapersonal psychosocial* *support*, *Interpersonal psychosocial* *support*, and *Institutional/systemic* *support*. *p*‐values < 0.1 were considered statistically significant.

For aim 3, we ran separate univariable logistic regression models for the outcomes of *intrapersonal*, *interpersonal*, and *institutional/systemic* psychosocial support needs during treatment and survivorship to investigate the associations with sociodemographic and cancer‐related characteristics. All characteristics that were statistically significant at *p* < 0.1 in the univariable model were included in the respective multivariable model (again one model for each outcome).

## 3 Results

### 3.1 Sample Characteristics

**FIGURE 1:**
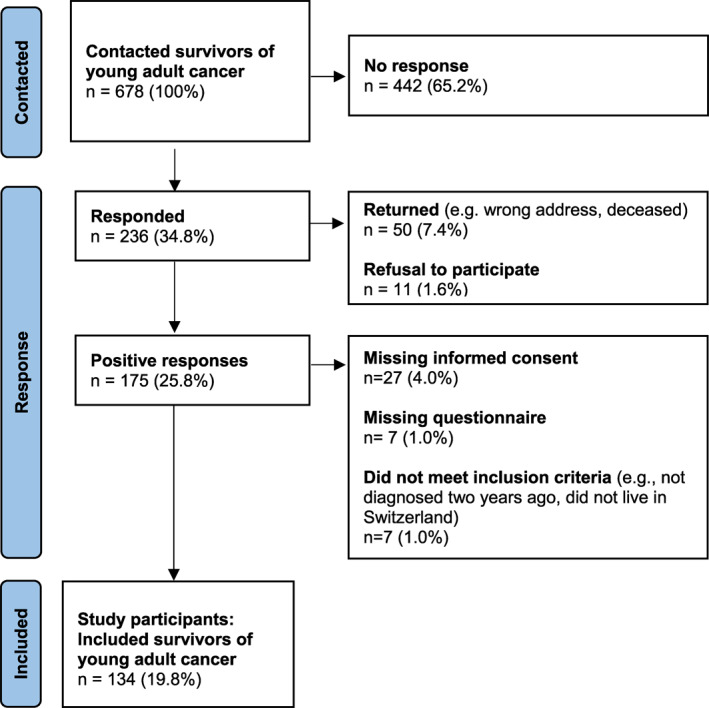
Study flow chart: Recruitment of survivors of young adult cancer and study participants.

We contacted 678 YACS and received 236 responses (34.8%). Of these, 175 (25.8%) were positive responses, and 134 participants (19.8%) were ultimately included in our analysis (Figure 1). The study population and the YACS population of Switzerland had similar gender and age distributions at diagnosis, but differed in terms of diagnosis, treatment, and second cancers (Supporting Information S1: Table S2). The study sample included a higher proportion of gonadal tumors (23.1% vs. 16%) and lymphomas (18% vs. 11%), and a higher proportion of melanomas (8% vs. 14%).A higher proportion of survivors had undergone chemotherapy (33% vs. 16%), radiotherapy (37% vs. 21%), and stem cell transplantation (7% vs. 1%). Additionally, a higher proportion of survivors in our study had experienced a second cancer (8% vs. 2%).

Participating survivors had a mean age of 37.5 years (SD = 5.1) at the time of the study (Table 1). Most survivors had completed upper secondary education (*n* = 53, 39.6%) and were employed full‐time (*n* = 78, 58.2%). Main diagnoses were carcinomas (*n* = 52, 38.8%), gonadal tumors (*n* = 31, 23.1%), and lymphomas (*n* = 24, 17.9%).

**TABLE 1:** Sociodemographic and cancer‐related characteristics of study participants.

	Survivors of young adult cancer (*n* = 134)		
	N	%	
*Sociodemographic characteristics*			
Gender			
Female	83	61.9	
Male	51	38.1	
Age at study (2023/4)			
21–30 years	13	9.7	
31–39 years	80	59.7	
≥ 40 years	41	30.6	
Migration background			
No	128	95.5	
Yes	6	4.5	
Educational achievement			
Compulsory schooling	3	2.2	
Vocational training	50	37.3	
Upper secondary education	53	39.6	
University education	28	20.9	
Employment status at study			
Employed full‐time	57	42.5	
Employed part‐time	35	26.1	
Homeworker	27	20.2	
Not employed	15	11.2	
Partnership at study			
Yes	105	78.4	
No	29	21.6	
*Cancer‐related characteristics*			
Diagnosis (Barr et al. 2020)^a^			
Carcinomas	52	38.8	
*Breast cancer*	*33*	*24.6*	
*Colorectal or anal cancer*	*12*	*8.9*	
*Thyroid cancer*	*5*	*3.7*	
*Pancreatic cancer*	*1*	*0.8*	
*Bladder cancer*	*1*	*0.8*	
Gonadal tumors	31	23.1	
Lymphomas	24	17.9	
Other tumors^b^	27	20.2	
Treatment^c^			
Surgery	31	23.1	
Chemotherapy	44	32.8	
Radiotherapy	50	37.3	
Stem cell transplantation	9	6.7	
Age at diagnosis			
21–30 years	51	38.1	
31–39 years	83	61.9	
Time since diagnosis			
2–5 years	56	41.8	
6–9 years	59	44.0	
10–13 years	19	14.2	
Late effects (self‐reported)			
No	89	66.4	
Yes	45	33.6	
Relapse			
No	121	90.3	
Yes	13	9.7	
Second cancer			
No	124	92.5	
Yes	10	7.5	
	Mean	SD	Range
Age at study (in years)	37.6	5.13	25–52
Age at diagnosis (in years)	31.3	5.03	21–40
Time since diagnosis (in years)	6.2	2.74	2–13

Abbreviations: CNS = central nervous system; *n* = number; SD = standard deviation.

^a^ Cancer categories according to the cancer classification by Barr et al. [22].

^b^ The category “other tumors” consists of leukemias, CNS neoplasms, sarcomas, nerve sheath tumors, and malignant melanoma.

^c^ Hierarchically coded: chemotherapy may include surgery, radiotherapy may include surgery and/or chemotherapy, stem cell transplantation may include surgery and/or chemotherapy and/or radiotherapy.

### 3.2 Aim 1: Description of the Psychosocial Support Needs in YACS

**FIGURE 2:**
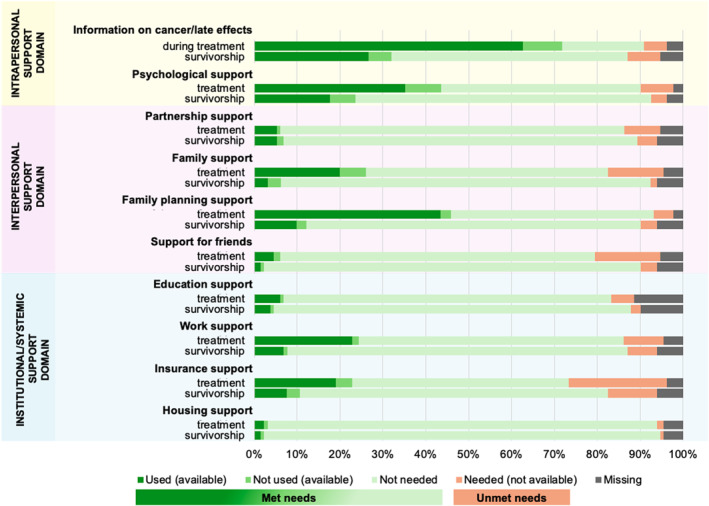
Psychosocial support needs of young adult cancer survivors for the different subcategories for need and the three categories for need during treatment and survivorship.

Most YACS reported *met needs* during both treatment and survivorship. Only a few reported *unmet needs* in the three support domains (Figure 2, more detailed: Supporting Information S1: Tables S3 and S4).

The most pronounced *met needs* during treatment were for housing support (*n* = 123, 94%), followed by family planning support (*n* = 122, 93%), and information on cancer and potential late effects (*n* = 120, 91%). During survivorship, the most prevalent *met need* was for housing support (*n* = 124, 95%), followed by psychological support (*n* = 121, 93%).

*Unmet needs* during treatment were most pronounced for insurance support (*n* = 30, 23%), followed by support for friends (*n* = 20, 15%) and family support (*n* = 17, 13%). During survivorship, the most pronounced *unmet needs* were for insurance support (*n* = 15, 12%), information about cancer and potential late effects (*n* = 10, 8%), and for work‐related support (*n* = 9, 7%).

Open answers revealed both the strengths and gaps in support (Supplementary information, Table S5). During treatment, YACS primarily expressed a need for assistance with physical side effects, psychological care and rehabilitation. They also mentioned the importance of addressing family‐related issues, such as childcare and fertility guidance, as well as bureaucratic matters, such as household support, and the need for peer contact. During survivorship, YACS reported unmet needs related to psychological care, rehabilitation, meetings with peers, and returning to work or leisure activities. Positive experiences during treatment included emotional support from family members, access to psychological care, peer contact, compassionate hospital care, and assistance from support services. During survivorship, YACS valued structured follow‐up care, support from cancer organizations, and emotional support from peers and loved ones.

### 3.3 Aim 2: Determining Changes in Unmet Psychosocial Support Needs

For unmet *intrapersonal* support needs, no statistically significant change was observed between treatment and survivorship (OR = 1.18; 95%CI = 0.49–2.91; *p* = 0.839; Table 2). The proportion of participants with *unmet needs* within the *interpersonal* support domain (OR = 6.75, 95%CI = 2.35–26.54; *p* < 0.001) and the *institutional/systemic* support domain (OR = 4.40, 95%CI = 1.63–14.87; *p* = 0.002) declined during survivorship compared to during treatment.

**TABLE 2:** Change in unmet needs for the three psychosocial support domains: intrapersonal, interpersonal and institutional/systemic support.

R			During survivorship		Odds Ratio (95% CI)	
			Unmet needs^d^ *n*	Met needs *n*		*p*‐value^f^
Intrapersonal support^a^	During treatment	Unmet needs^d^	4	13	1.18 (0.49–2.91)	0.839
		Met needs^e^	11	101		
Interpersonal support^b^		Unmet needs^d^	10	27	6.75 (2.35–26.54)	< 0.001
		Met needs^e^	4	88		
Institutional/systemic support^c^		Unmet needs^d^	15	22	4.40 (1.63–14.87)	0.002
		Met needs^e^	5	87		

*Note: p*‐values < 0.05 are indicated in bold font.

Abbreviations: *n* = Number, CI = Confidence interval.

^a^ Intrapersonal support includes information on cancer and late effects, and the psychological support.

^b^ Interpersonal support includes support on family, partnership, friends, and family planning.

^c^ Institutional/systemic support needs include support on education, work, insurance, and housing situation.

^d^ Unmet needs include the category: needed (not available).

^e^ Met needs include the categories: used (available), not used (available), and not needed.

^f^ Exact McNemar significance probability.

### 3.4 Aim 3: Associated Factors for Unmet Needs

Multivariable logistic regression analyses showed that male sex was significantly associated with more *interpersonal* support needs during treatment (OR = 2.51, 95% CI, 1.06–5.93, *p* = 0.036; Table 3). Younger age at study (21–30 vs. ≥ 31 years; OR = 0.23, 95% CI, 0.05–0.94, *p* = 0.003) and shorter time since diagnosis (2–5 vs. 6–13 years; OR = 0.20, 95% CI, 0.05–0.69, *p* = 0.04) were significantly associated with more *intrapersonal* support needs during survivorship.

**TABLE 3:** Unmet intrapersonal, interpersonal, and institutional/systemic psychosocial support needs during treatment and survivorship and their associations with sociodemographic and cancer‐related characteristics.

	Univariable logistic regression						Multivariable logistic regression					
	Intrapersonal support domain											
	During treatment *n* = 132			During survivorship *n* = 129			During treatment —			During survivorship *n* = 129		
	OR	95% CI	*p*‐value	OR	95% CI	*p*‐value	OR	95% CI	*p*‐value	OR	95% CI	*p*‐value
Sociodemographic characteristics												
**Sex** Female Male	Ref. 3.111	[0.85, 11.43]	**0.087**	Ref. 1.211	[0.39, 3.78]	0.741						
**Age at study** 21–30 years ≥ 31 years	Ref. 1.864	[0.23, 15.33]	0.562	Ref. 0.235	[0.06, 0.89]	**0.033**				Ref. 0.230	[0.05, 0.94]	**0.003**
**Educational achievement** **^a^** Low High	Ref. 0.885	[0.31, 2.50]	0.318	Ref. 2.017	[0.43, 9.50]							
**Employment status** ^b^ Employed Other situation	Ref. 1.600	[0.56, 4.55]	0.378	Ref. 1.569	[0.52, 4.75]	0.426						
**Partnership** Yes No	Ref. 5.150	[0.65, 40.59]	0.120	Ref. 2.017	[0.43, 9.50]	0.375						
Cancer‐related characteristics												
**Cancer diagnosis** Carcinomas Gonadal tumors Lymphomas Other tumors^c^	Ref. 0.671 1.88 2.82	[0.12, 3.69] [0.46, 7.74] [0.77, 10.32]	0.254	Ref. 1.128 1.1 0.611	[0.29, 4.37] [0.25, 4.85] [0.11, 0.32]	0.903						
**Treatment** ^d^ Surgery Chemotherapy Radiotherapy and stem cell transplantation	Ref. 1.23 0.75	[0.33, 4.63] [0.19, 2.89]	0.706	Ref. 0.923 1.5	[0.19, 4.46] [0.37, 6.13]	0.710						
**Age at diagnosis** 21–30 years 31–40 years	Ref. 0.511	[0.18, 1.43]	0.200	Ref. 0.942	[0.31, 2.83]	0.916						
**Time since diagnosis** 2–5 years 6–13 years	Ref. 1.361	[0.47, 3.92]	0.565	Ref. 0.204	[0.06,0.68]	**0.006**				Ref. 0.201	[0.05, 0.69]	**0.004**
**Late effects** No Yes	Ref. 1.418	[0.50, 4.02]	0.514	Ref. 1.821	[0.61, 5.40]	0.284						
	**Interpersonal support domain**											
	During treatment *n* = 131			During survivorship *n* = 130			During treatment *n* = 131			During survivorship		
	OR	95% CI	*p*‐value	OR	95% CI	*p*‐value	OR	95% CI	*p*‐value			
Sociodemographic characteristics												
**Sex** Female Male	Ref. 2.432	[1.04, 5.71]	**0.034**	Ref. 0.785	[0.26, 2.42]	0.675	Ref. 2.508	[1.06, 5.93]	**0.036**			
**Age at study** 21–30 years 31‐≥ 41 years	Ref. 1.406	[0.36, 5.42]	0.612	Ref. 1.362	[0.16, 11.42]	0.767						
**Educational achievement** **^a^** Low High	Ref. 1.852	0.82, 4.17]	0.129	Ref. 1.140	[0.36, 3.62]	0.822						
**Employment status** ^b^ Employed Other situation	Ref. 1.680	[0.76, 3.71]	0.199	Ref. 2.412	[0.79, 7.40]	0.124						
**Partnership** Yes No	Ref. 0.882	[0.36, 2.16]	0.786	Ref. 1.059	[0.27, 4.08]	0.933						
Cancer‐related characteristics												
**Cancer diagnosis** Carcinomas Gonadal tumors Lymphomas Other tumors^c^	Ref. 0.547 0.901 1.158	[0.19, 1.59] [0.32, 2.60] [0.43, 3.15]	0.616	Ref. 0.643 1.286 1.636	[0.12, 3.54] [0.28, 5.89] [0.39, 6.70]	0.748						
**Treatment** ^d^ Surgery Chemotherapy Radiotherapy and stem cell transplantation	Ref. 3.232 3.044	[0.95, 11.01] [0.93, 10.00]	**0.096**	Ref. 0.923 1.5	[0.19, 4.46] [0.36, 6.13]	0.710	Ref. 1.283	[0.58, 2.83]	0.535			
**Age at diagnosis** 21–30 years 31–40 years	Ref. 0.829	[0.38, 1.79]	0.635	Ref. 1.141	[0.36, 3.62]	0.822						
**Time since diagnosis** 2–5 years 6–13 years	Ref. 1.157	[0.54, 2.50]	0.709	Ref. 0.492	[0.16, 1.51]							
**Late effects** No Yes	Ref. 0.879	[0.39, 1.97]	0.754	Ref. 2.135	[0.69, 6.53]	0.187						
	**Institutional/systemic support domain**											
	During treatment *n* = 131			During survivorship *n* = 130			During treatment			During survivorship		
	OR	95% CI	*p*‐value	OR	95% CI	*p*‐value						
Sociodemographic characteristics												
**Sex** Female Male	Ref. 0.988	[0.45, 2.16]	0.975	Ref. 1.103	[0.68, 5.89]	0.192						
**Age at study** 21–30 years 31‐≥ 41 years	Ref. 0.434	[0.14, 1.39]	0.167	Ref. 2.111	[0.26, 17.33]	0.446						
**Educational achievement** **^a^** Low High	Ref. 1.149	[0.67, 3.23]	0.317	Ref. 1.37	[0.50, 3.76]	0.543						
**Employment status** ^b^ Employed Other situation	Ref. 1.148	[0.52, 2.56]	0.736	Ref. 1.204	[0.44, 3.29]	0.717						
**Partnership** Yes No	Ref. 0.589	[0.25, 1.40]	0.239	Ref. 1.753	[0.47, 6.46]	0.375						
Cancer‐related characteristics												
**Cancer diagnosis** Carcinomas Gonadal tumors Lymphomas Other tumors^c^	Ref. 2.567 1.729 2.62	[0.93, 7.11] [0.57, 5.30] [0.92, 7.49]	0.191	Ref. 1.229 2.048 0.512	[0.35, 4.28] [0.60, 6.95] [0.07, 0.36]	0.391						
**Treatment** ^d^ Surgery Chemotherapy Radiotherapy and stem cell transplantation	Ref. 1.358 0.916	[0.49, 3.79] [0.34, 2.50]	0.654	Ref. 1.842 4.136	[0.33, 10.19] [0.87, 19.73]	**0.091**						
**Age at diagnosis** 21–30 years 31–40 years	Ref. 0.712	[0.33, 1.53]	0.385	Ref. 1.556	[0.56, 4.36]	0.391						
**Time since diagnosis** 2–5 years 6–13 years	Ref. 1.52	[0.69, 3.33]	0.294	Ref. 0.85	[0.32, 2.21]	0.733						
**Late effects** No Yes	Ref. 1.167	[0.53, 2.56]	0.702	Ref. 1.37	[0.51, 3.65]	0.531						

*Note:* Statistically significant variables at *p* < 0.1 are highlighted in bold.

Abbreviations: CI = confidence interval, CNS = central nervous system, OR = odds ratio, ref = reference category, *n* = Numbers.

^a^ Educational achievement: low includes compulsory schooling and vocational training; high includes upper secondary education and university education.

^b^ Employment status at study: Employed includes full‐time and part‐time employment. Other situations included homeworkers and those not employed.

^c^ The category “other tumors” consists of leukemias, CNS neoplasms, sarcomas, nerve sheath tumors, and malignant melanoma.

^d^ Treatment was coded hierarchically as “surgery only”; “chemotherapy (may have had surgery)”; radiotherapy and stem cell transplantation (may have had surgery and/or chemotherapy)”.

## 4 Discussion

With this study, we aimed to better understand the psychosocial support needs of YACS in Switzerland, changes in needs between treatment and survivorship, and associations with socio‐demographic and cancer‐related characteristics. Overall, psychosocial support needs were met for most survivors across *intrapersonal*, *interpersonal*, and *institutional/systemic* support domains. During treatment, unmet needs were highest for insurance support and support for friends and family, whereas during survivorship, they were most pronounced for insurance, information on cancer and late effects, and work‐related support. *Intrapersonal* support needs remained stable from treatment to survivorship, while unmet *interpersonal* and *institutional/systemic* support needs declined during survivorship. Male sex was associated with more *interpersonal* support needs during treatment. Younger age at diagnosis and shorter time since diagnosis were associated with more *intrapersonal* support needs during survivorship.

The high proportion of met needs likely reflects the integration of multidisciplinary medical and psychosocial services within Swiss hospitals. As demonstrated in previous research, the most prevalent support needs among (A)YAs with cancer pertained to aspects of their disease and treatment, as well as possible late effects (e.g., the possible consequences of the illness) [23, 24]. Consistent with these findings, our study identified that unmet needs related to information concerning cancer and potential late effects constituted the second most frequently reported subdomain during survivorship.

Longitudinal studies have indicated that while certain psychosocial and interpersonal unmet needs may diminish following treatment, others persist throughout survivorship [25, 26]. Consistent with these findings, our results suggest that interpersonal unmet needs generally decrease from treatment to survivorship, while support for friends and family remains insufficient, thereby underscoring the persistent social challenges experienced by AYA cancer survivors. Typically, (A)YAs rely on a variety of support systems, including partners, family members, friends, or peers. The emotional and practical demands placed on these informal caregivers can be substantial. Studies have shown that this burden may lead to physical and psychological burdens in these caregivers [27, 28]. While caregiver‐specific services are not consistently embedded in routine Swiss oncology care, there are structured resources available. These include caregiver‐focused support groups, peer networks, and counseling offered by the Swiss Cancer League and regional programs. However, these services are generally not tailored specifically to the AYA cancer population [29, 30, 31]. The provision of tailored information to friends, peers, and family members of YACS could not only improve the survivors' reintegration, but also help prevent secondary emotional burdens among their close social circles.

Within the institutional/systemic domain, insurance‐related unmet needs were prominent. Although basic health insurance is mandatory in Switzerland, access to supplementary or disability insurance varies and may involve complex and stressful application processes. A similar description of the disability application processes was reported by Swiss childhood cancer survivors who described these processes as complex, burdensome, and emotionally distressing [32]. Some YACS reported that they would have wished for more financial support or coverage of fertility treatments. A recent Swiss study revealed that insurance coverage for fertility preservation procedures is inconsistent, and that clear, country‐specific information is required [33]. A systematic review of studies on survivors of cancer in childhood and adolescence showed that insurance‐related challenges included difficulties obtaining and maintaining insurance, higher premiums, and financial distress due to medical expenses [34].

Our findings revealed that a considerable number of YACS selected the category “not used (available)”, particularly within the intrapersonal domain concerning information on cancer and late effects, as well as psychological support, during both treatment and survivorship. This was interpreted as a “met need”, although the reason for non‐use remains unclear. The present study's participants were treated at a Swiss hospital, where they were offered medical, but also psychosocial support and care throughout their treatment. Previous research suggests that barriers to psychosocial care are multifactorial, including personal factors (e.g., lack of motivation, feeling too unwell, or practical challenges), as well as service‐related factors (e.g., lack of availability, inappropriate support, or lack of accessibility), and systemic issues (e.g., not offered at all, lack of integration, or inconsistency) [35]. This may help to explain our results, particularly as some participants also expressed the need for lower thresholds to access psychological or rehabilitation therapy during treatment in open responses. As our findings further suggest that support needs change throughout the cancer continuum, we concur with the recommendation from Holland et al. and Lau et al. to continue offering support services at multiple time points to address changing needs over time [35, 36].

Male survivors reported higher intrapersonal and interpersonal support needs during treatment, consistent with previous findings showing greater unmet informational and emotional needs and lower help‐seeking behavior among men [37, 38]. One potential explanation for this is that young men with cancer encounter unique psychosocial challenges that are shaped by cultural expectations of masculinity. These expectations may act as barriers to the seeking and receipt of emotional support, as men may feel compelled to appear strong and self‐reliant [39].

Our results indicated that younger age at study and shorter time since diagnosis were significantly associated with more intrapersonal support needs during survivorship. Compared to older young adults, young adults aged 21–30 years may face greater challenges regarding their dependence on parents, establishing life goals, and forming an identity after cancer [40]. There are mixed findings in the literature regarding the association between time since diagnosis and informational needs [41, 42]. One potential explanation for our finding is that individuals closer to their diagnosis may be more likely to experience feelings of anxiety and uncertainty, which could in turn lead to increased intrapersonal support needs, such as psychological support and information about late effects.

### 4.1 Limitations and Strengths

This is the first study in Switzerland to assess the support needs during both treatment and survivorship in YACS. The study benefited from collaborating with four different Swiss clinics, which facilitated participant recruitment. A wide range of support domains (10 subdomains) were assessed in detail using nuanced response categories. These captured not only whether a service was needed or used, but also whether it was available but ultimately not needed. This allowed for a more differentiated understanding of support utilization. Additionally, the few additional topics arising from the open‐ended answers indicate that our questionnaire adequately covered psychosocial support needs. Alongside the study's strengths, there are also limitations to acknowledge. Firstly, the sample size was relatively small, and the sample was limited to German‐speaking participants, which may reduce the generalizability of the findings to other language regions within Switzerland. Despite our sample being similar to the Swiss YACS population regarding gender and age at diagnosis, substantial differences in diagnosis, treatment, and second cancer history may affect the transferability of our results to the broader Swiss survivor population. The higher proportion of YACS with gonadal tumors, more intensive treatments (e.g., chemotherapy, radiotherapy, stem cell transplantation), and second tumors in our sample likely indicates a greater overall medical and psychosocial burden, which may lead to different or greater psychosocial needs [43, 44, 45]. Although including four clinics facilitated recruitment, the number of participating institutions was limited due to legal and organizational hurdles, restricting broader outreach and potentially introducing selection bias. Secondly, the study did not assess the sources from which participants received support, such as healthcare professionals, patient organizations, or informal networks. This limits our understanding of how support systems function in practice. However, some open‐ended responses did mention specific institutions in a positive light. It should also be considered that survivors' or their families' financial situation might have influenced their access to, or perception of support. This aspect was not examined in this study, but would require further investigation. Thirdly, we did not ask participants how they would have preferred support to be delivered (e.g., in person, anonymously, or via digital platforms), which restricts the conclusions that can be drawn about the accessibility or acceptability of services. Moreover, the classification of response categories, particularly *not used (available)*, posed interpretative challenges. While coded as a *met need,* this category may also reflect partially *unmet needs*, since no additional details were available to clarify the reasons for non‐use. Furthermore, only 5% of our sample had a migration background, compared to approximately 27% in the Swiss general population [46]. This difference, which has also been observed in other studies, may be related to language or participation factors, and should be considered when evaluating the generalizability of the findings [46]. Finally, support needs were assessed retrospectively, which may have introduced recall bias, particularly when distinguishing between needs during treatment and survivorship.

### 4.2 Clinical Implications

This study provides a foundation for important clinical implications regarding the provision of support services for AYAs with cancer during treatment and survivorship in Switzerland.

1. **Continued and appropriate support along the cancer continuum:** Although most support needs were met, our results showed that certain needs persisted or emerged during survivorship, particularly in the areas of *interpersonal* and *institutional/systemic* support. This underscores the importance of providing psychosocial support throughout survivorship, not just during acute treatment.
2. **Addressing unmet institutional/systemic needs**: Participants reported unmet needs relating to insurance, especially during treatment, and highlighted gaps in financial and fertility treatment coverage. Given the complexity of the Swiss health insurance system, particularly with regard to supplementary and life insurance, providing clear, timely, and tailored guidance could help survivors to navigate these issues. Offering such counseling early on and at key transition points (e.g., from treatment to follow‐up care) could be beneficial.
3. **Enhancing access to psychosocial support:** A considerable proportion of participants selected the response “not used (available)” in the intrapersonal domain, particularly for psychological support. This may indicate barriers to access, rather than an absence of support offers in principle. Efforts should be made to reduce these barriers, for instance by offering flexible delivery modes (e.g., online, anonymous, or with low‐threshold entry points), and improving communication about the availability and benefits of such services.
4. **Involving the social environment (**e.g.**, friends and family):** Unmet interpersonal needs during survivorship, particularly concerning support for friends and family, suggest a lack of information for or involvement from their social environment. This highlights the potential value of ensuring that informal caregivers also have access to relevant information, especially during long‐term survivorship when contact with the healthcare team is less frequent and support needs may change. Rather than creating new materials, efforts should be made to make existing information easier to find, understand, and apply in everyday life.
5. **Gender‐sensitive support approaches:** Our study found that male survivors reported significantly higher needs for interpersonal support during treatment. Open answers revealed a desire for male‐specific support services during survivorship. These findings emphasize the need for psychosocial care models that are sensitive to gender and acknowledge masculinity‐related help‐seeking behaviors and offer tailored services to male survivors.

Taken together, these findings highlight the need for continuous, accessible, and personalized psychosocial care across the cancer care continuum. Support services should be age‐appropriate, gender‐specific, holistic, and, responsive to changing needs from diagnosis through treatment and into survivorship [47]. To ensure this, such services should be available at key points and offered repeatedly and proactively throughout the entire course of care.

## 5 Conclusion

This study examined the support needs of survivors of young adult cancer in Switzerland, spanning the treatment and survivorship phases. While most support needs were met, persistent and emerging unmet needs, particularly in the *interpersonal* and *institutional/systemic* support domains, underscore the importance of continuous, personalized psychosocial support. Notably, challenges regarding access to psychological support and insurance support, as well as support for friends of survivors, require targeted assistance. Gender‐specific patterns emerged, such as male survivors reporting greater interpersonal support needs, which further emphasizes the need for more inclusive information and support. Future services should build on these insights to ensure continuity, responsiveness, and equity in (A)YA survivorship care.

## Author Contributions

conceptualization: K.R. methodology, K.R. and C.B. formal analysis, C.B. investigation, O.G., W.M., B.M., M.V., K.R., and C.B. resources, M.B., L.M., M.M., and K.R. data curation, C.B., M.O., and K.R. writing–original draft preparation, C.B. writing review and editing, all authors. visualization, C.B. supervision, K.R. project administration, K.R. and C.B. funding acquisition, K.R. All authors have read and agreed to the published version of the manuscript.

## Funding

This work was supported by Palatin‐Stiftung Switzerland (Nr. 0028/2020), Krebsliga Zentralschweiz Switzerland, and Avenira Stiftung Switzerland.

## Ethics Statement

All procedures performed in this study were in accordance with the ethical standards of the responsible research committee and with the 1964 Helsinki Declaration and its later amendments or comparable ethical standards. The study was approved by the Ethics Committee Northwest and Central Switzerland (EKNZ 2022–01065, 12 October 2022).

## Consent

Written informed consent on paper was obtained from all individual participants included in the study.

## Conflicts of Interest

The authors declare no conflicts of interest.

## Supporting information


Supporting Information S1

## Data Availability

The data that support the findings of this study are available from the corresponding author upon reasonable request.
