# Supplementary material for: Psychosocial Support Needs and Utilization of Support Among Survivors of Cancer in Young Adulthood in Switzerland: A Report From the Adolescent and Young Adult (AYA) Psychosocial Health Study
Source: Psychooncology. 2026 Feb 10;35(2):e70399. doi: 10.1002/pon.70399 (PMC12888951; doi:10.1002/pon.70399)
Supplement: Supplementary file 1 — Supporting Information S1 [file PON-35-e70399-s001.docx]

[Psycho-Oncology]

Appendix to the manuscript

**Psychosocial support needs and utilization of support among survivors of cancer in young adulthood in Switzerland:**

**A report from the AYA Psychosocial Health Study**

Céline Bolliger^1^, Martina Ospelt^1^, Marcel Blum^2^, Oliver Gautschi^3^, Luzius Mader^4^, Walter Mingrone^5^, Mohsen Mousavi^2^, Beat Müller^3^, Marcus Vetter^6^, Katharina Roser^1*^

^1^ Faculty of Health Sciences and Medicine, University of Lucerne, Lucerne, Switzerland

^2^ Cancer Registry of Eastern Switzerland, St. Gallen, Switzerland

^3^ Department for Medical Oncology, Cantonal Hospital of Lucerne, Lucerne, Switzerland

^4^ Cancer Registry Bern and Solothurn, Bern, Switzerland

^5^ Department for Oncology, Cantonal Hospital Olten, Olten, Switzerland

^6^ Center for Oncology and Hematology, University Hospital Basel, Basel, Switzerland

* Correspondence: [katharina.roser@unilu.ch](mailto:katharina.roser@unilu.ch); Tel.: +41 41 229 59 56

Overview

Supplementary Information 1 – Table S1: Questionnaire on psychosocial support in young adult cancer survivors

Supplementary Information 2 – Table S2: Comparison of YACS participants and the Swiss 2-year YACS population

Supplementary Information 3 – Table S3: Psychosocial support needs of young adult cancer survivors (detailed need subcategories)

Supplementary Information 4 – Table S4: Psychosocial support needs of young adult cancer survivors (main need categories)

Supplementary Information 5 – Table S5: Summary of coded answers to open-ended questions

**Supplementary Information 1 – Table S1: Questionnaire on psychosocial support in young adult cancer survivors**

**Table S1**. Questionnaire on psychosocial support in survivors of young adult cancer (self-developed and translated into English).

| **Psychosocial support** |
| --- |

| 1. Please consider the following services and to what extent you **made use of them** or **would have wished** to. Please refer both to the time during your cancer treatment (highlighted in beige) and to the present (highlighted in green). |
| --- |

|  | **During cancer treatment** | | | | | **Today (during survivorship)** | | | | |
| --- | --- | --- | --- | --- | --- | --- | --- | --- | --- | --- |
|  | I used it | It was available, I could have used it, but did not | It was available, but I did not need it | It was not available, but I would have been interested | I did not need it | I am using it | It is available, I could use it, but I still do not | It is available, but I do not need it | It is not available, but I would be interested | I do not need it |
| **Information** about **cancer** and potential late effects | 🗆 1 | 🗆 2 | 🗆 3 | 🗆 4 | 🗆 5 | 🗆 1 | 🗆 2 | 🗆 3 | 🗆 4 | 🗆 5 |
| **Psychological** support | 🗆 1 | 🗆 2 | 🗆 3 | 🗆 4 | 🗆 5 | 🗆 1 | 🗆 2 | 🗆 3 | 🗆 4 | 🗆 5 |
| Support for your **education** (e.g., school, during vocational training, or at university) | 🗆 1 | 🗆 2 | 🗆 3 | 🗆 4 | 🗆 5 | 🗆 1 | 🗆 2 | 🗆 3 | 🗆 4 | 🗆 5 |
| Support with your **professional situation** (e.g., adjusting working hours, job search) | 🗆 1 | 🗆 2 | 🗆 3 | 🗆 4 | 🗆 5 | 🗆 1 | 🗆 2 | 🗆 3 | 🗆 4 | 🗆 5 |
| Support with **insurance** (e.g., questions about health insurance, disability insurance) | 🗆 1 | 🗆 2 | 🗆 3 | 🗆 4 | 🗆 5 | 🗆 1 | 🗆 2 | 🗆 3 | 🗆 4 | 🗆 5 |
| Support with your housing situation (e.g., finding an apartment) | 🗆 1 | 🗆 2 | 🗆 3 | 🗆 4 | 🗆 5 | 🗆 1 | 🗆 2 | 🗆 3 | 🗆 4 | 🗆 5 |
| Support for your **partnership** (e.g., support in your current relationship) | 🗆 1 | 🗆 2 | 🗆 3 | 🗆 4 | 🗆 5 | 🗆 1 | 🗆 2 | 🗆 3 | 🗆 4 | 🗆 5 |
| Support for your family (e.g. providing information for your family) | 🗆 1 | 🗆 2 | 🗆 3 | 🗆 4 | 🗆 5 | 🗆 1 | 🗆 2 | 🗆 3 | 🗆 4 | 🗆 5 |
| Support for your **family planning** and/or **desire to have children** (e.g., questions about fertility and having children) | 🗆 1 | 🗆 2 | 🗆 3 | 🗆 4 | 🗆 5 | 🗆 1 | 🗆 2 | 🗆 3 | 🗆 4 | 🗆 5 |
| Support for your **friends** (e.g., providing information for your friends) | 🗆 1 | 🗆 2 | 🗆 3 | 🗆 4 | 🗆 5 | 🗆 1 | 🗆 2 | 🗆 3 | 🗆 4 | 🗆 5 |

| 1. Other/additional areas where you would have wished for support **during** your **cancer treatment**: |
| --- |

🖊

| 1. Other/additional areas where you wish to receive support **today** (during survivorship): |
| --- |

🖊

______________________________________________________________________________________________________________

**Supplementary Information 2 – Table S2: Comparison of YACS participants and the Swiss 2-year YACS population**

**Table S2**. Comparison of the YACS population of the Psychosocial Health Study and the Swiss 2-year YACS population.

|  | **Young adult cancer survivors (YACS)** | | | | | | |
| --- | --- | --- | --- | --- | --- | --- | --- |
|  | **YACS Population of the Psychosocial Health Study^a^** | | **YACS 2-year Population of Switzerland^b^** | | | | **Chi^2^ test** |
|  | **Observed** | | **Observed**^c^ | | **Estimated**^d^ | |  |
|  | N=134 | | N=13’290 | | N=15’603.3 | |  |
|  | **n** | **%** | **n** | **%** | **n** | **%** | **p-value** |
| Sex  Male  Female | 51  83 | 38.1  61.9 | 5’704 7’586 | 42.9 57.1 | 6708.1  8895.1 | 43.0  57.0 | 0.251 |
| Age at diagnosis  20-24^a^  25-29  30-34  35-39 | 16  28  50  40 | 11.9  20.9  37.3  29.9 | 1’245 2’504 4’011 5’530 | 9.4 18.8 30.2 41.6 | 1’476.2 2’948.6 4’692.4 6’486.0 | 9.5 18.9 30.1 41.6 | 0.049 |
| Diagnosis (according to Barr et al. 2020)   1. Leukemias and related disorders 2. Lymphomas 3. CNS and other intracranial/intraspinal neoplasms 4. Sarcomas 5. Blood and lymphatic vessel tumor^e^ 6. Nerve sheath tumors 7. Gonadal and related tumors 8. Melanoma, malignant 9. Carcinomas 10. Miscellaneous specified neoplasms^e^ 11. Unspecified malignant neoplasms except CNS^e^ | 5  24  7  3  -  1  31  11  52  -  - | 3.7  17.9  5.2  2.2  -  0.8  23.1  8.2  38.8  -  - | 541  1’412  1’121  440  79  153  2’605  1’923  5’500  24  32 | 4.1  10.6  8.4  3.3  0.6  1.2  15.5  14.5  41.4  0.2  0.2 | 633.9  1’651.7  1’326.4  516.7  92.2  1’81.3  2’443.4  2’248.7  6’443.0  28.2  37.8 | 4.1  10.6  8.5  3.3  0.6  1.2  15.7  14.4  41.3  0.2  0.2 | **0.015** |
| Treatment^f^  Surgery only  Chemotherapy  Radiotherapy  Stem cell transplantation | 31  44  50  9 | 23.1  32.8  37.3  6.7 | 5’277  2’101  2’832  101 | 39.7  15.8  21.3  0.8 | 6’253  2’491  3’326  118 | 40.1  16.0  21.3  0.8 | **p<0.001** |
| Second cancer^d^  No  Yes | 124  10 | 92.5  7.5 | 12’876  202 | 98.5  1.5 | 15129.3  231.3 | 98.5  1.5 | **p<0.001** |

Abbreviations: YACS = Young adult cancer survivor; CNS = Central nervous system

Note: We conducted the chi-square test with the estimated number of cancer cases in the YA population of Switzerland.

^a^ The YA population from the psychosocial health study includes individuals diagnosed between the ages of 21 to 39 years (inclusion criteria)

^b^ The YA population of Switzerland includes individuals diagnosed with cancer between the ages of 20-39 years, and the initial cancer diagnosis was between 1980 and 2019. Data covers the whole of Switzerland, and participants were registered in the Swiss Childhood Cancer Registry (ChCR, age 15-19 years) and at the National Agency for Cancer Registration (NACR, age 20-39 years), respectively. Furthermore, the YA population was restricted to at least 2 years after diagnosis.

^c^ Observed: number of cancer cases collected by the ChCR and/or the NACR. Covered 63% of the AYA cancers diagnosed in Switzerland between 1980-2019.

^d^ Estimated: number of cases after extrapolation of the observed cancer cases to the whole of Switzerland to acquire 100% coverage. Characteristic-specific totals don’t necessarily add up to the same estimated column total.

^e^ Cancer cases in these three cancer categories from Barr et al. 2020 – (5) Blood and lymphatic vessel tumors, (10) Miscellaneous specified neoplasms, and (11) Unspecified malignant neoplasms except CNS –have been recorded under different categories.

^f^ Hierarchically coded: chemotherapy may include surgery, radiotherapy may include surgery and/or chemotherapy, stem cell transplantation may include surgery and/or chemotherapy and/or radiotherapy. Those don’t add up to the column total for the populations of the epidemiological study, because of cancer cases that received other treatments or had unknown treatment.

**Supplementary Information 3 – Table S3: Psychosocial support needs of young adult cancer survivors (detailed need subcategories)**

**Table S3**. Psychosocial support needs of young adult cancer survivors for each subcategory of need during treatment and survivorship.

| **Support area** | | **During treatment** | | | | | **During survivorship** | | | | |
| --- | --- | --- | --- | --- | --- | --- | --- | --- | --- | --- | --- |
|  |  | **Used (available)** | **Not used (available)** | **Not needed** | **Needed (not available)** | **Missing values** | **Used (available)** | **Not used (available)** | **Not needed** | **Needed (not available)** | **Missing values** |
|  |  | **n (%)** | **n (%)** | **n (%)** | **n (%)** | **n (%)** | **n (%)** | **n (%)** | **n (%)** | **n (%)** | **n (%)** |
| Intrapersonal support domain^a^ | Information about cancer / potential late effects | 82 (62.6) | 12 (9.2) | 26 (19.1) | 7 (5.3) | 5 (3.8) | 35 (26.7) | 7 (5.3) | 72 (55.0) | 10 (7.6) | 7 (5.3) |
|  | Psychological support | 46 (35.1) | 11 (8.4) | 61 (46.5) | 10 (7.6) | 3 (2.3) | 22 (17.8) | 8 (6.1) | 91 (69.5) | 5 (3.8) | 5 (3.8) |
| Interpersonal support domain^b^ | Partnership support | 7 (5.3) | 1 (0.8) | 105 (80.2) | 11 (8.4) | 7 (5.3) | 7 (5.3) | 2 (1.5) | 108 (82.4) | 6 (4.5) | 8 (6.1) |
|  | Family support | 26 (19.9) | 8 (6.1) | 74 (56.5) | 17 (13.0) | 6 (4.6) | 4 (3.1) | 4 (3.1) | 113 (86.3) | 2 (1.5) | 8 (6.1) |
|  | Family planning support | 57 (43.5) | 3 (2.3) | 62 (47.3) | 6 (4.6) | 3 (2.3) | 13 (9.9) | 3 (2.3) | 102 (77.9) | 5 (3.8) | 8 (6.1) |
|  | Support for friends | 6 (4.56) | 2(1.5) | 96 (73.3) | 20 (15.3) | 7 (5.3) | 2 (1.5) | 1 (0.8) | 115 (87.8) | 5 (3.8) | 8 (6.1) |
| Institutional / systemic support domain^c^ | Educational support | 8 (6.1) | 1 (0.8) | 100 (76.3) | 7 (5.3) | 15 (11.5) | 5 (3.8) | 1 (0.8) | 109 (83.2) | 3 (2.3) | 13 (9.9) |
|  | Work-related support | 30 (22.9) | 2 (1.5) | 81 (61.8) | 12 (9.2) | 6 (4.6) | 9 (6.9) | 1 (0.8) | 111 (84.8) | 9 (6.9) | 8 (6.1) |
|  | Insurance support | 25 (19.1) | 5 (3.8) | 66 (50.4) | 30 (22.9) | 5 (3.8) | 10 (7.6) | 4 (3.1) | 94 (71.8) | 15 (11.5) | 8 (6.1) |
|  | Housing support | 3 (2.3) | 1 (0.8) | 119 (90.9) | 2 (1.5) | 6 (4.6) | 2 (1.5) | 1 (0.8) | 121 (92.4) | 1 (0.8) | 6 (4.6) |

Abbreviations: n = Number

^a^ Intrapersonal support includes information on cancer and late effects, and psychological support.

^b^ Interpersonal support includes support for family, partnership, friends, and family planning.

^c^ Institutional/systemic support includes support for education, work, insurance, and housing situation.

**Supplementary Information 4 – Table S4: Psychosocial support needs of young adult cancer survivors (main need categories)**

Table S4. Unmet and met psychosocial support needs of young adult cancer survivors during treatment and survivorship.

| **Support area** | | **During treatment** | | | **During survivorship** | | |
| --- | --- | --- | --- | --- | --- | --- | --- |
|  |  | **Unmet needs^a^** | **Met needs^b^** | **Missing values** | **Unmet needs^a^** | **Met needs^b^** | **Missing values** |
|  | | **n (%)** | **n (%)** | **n (%)** | **n (%)** | **n (%)** | **n (%)** |
| Intrapersonal support domain | Information about cancer / potential late effects | 7 (5.3) | 120 (90.9) | 5 (3.8) | 10 (7.6) | 114 (87.0) | 7 (5.2) |
|  | Psychological support | 10 (7.6) | 118 (90.0) | 3 (2.3) | 5 (3.8) | 121 (93.4) | 5 (3.7) |
| Interpersonal support domain | Partnership support | 11 (8.4) | 113 (86.3) | 7 (5.3) | 6 (4.5) | 117 (89.2) | 13 (9.7) |
|  | Family support | 17 (13.0) | 108 (82.5) | 6 (4.6) | 2 (1.5) | 121 (92.5) | 8 (6.0) |
|  | Family planning support | 6 (4.6) | 122 (93.1) | 3 (2.3) | 5 (3.8) | 118 (90.1) | 8 (6.0) |
|  | Support for friends | 20 (15.3) | 104 (79.4) | 7 (5.3) | 5 (3.8) | 118 (90.1) | 6 (4.5) |
| Institutional/systemic support domain | Educational support | 7 (5.3) | 109 (83.2) | 15 (11.5) | 3 (2.3) | 115 (87.8) | 8 (6.0) |
|  | Work-related support | 12 (9.2) | 113 (86.2) | 6 (4.6) | 9 (6.9) | 121 (92.5) | 8 (6.0) |
|  | Insurance support | 30 (22.9) | 96 (73.3) | 5 (3.8) | 15 (11.5) | 108 (82.5) | 8 (6.0) |
|  | Housing support | 2 (1.5) | 123 (94.0) | 6 (4.6) | 1 (0.8) | 124 (94.7) | 8 (6.0) |

^a^ Unmet needs include the category: needed (not available).

^b^ Met needs include the categories: used, not used (available), and not needed.

**Supplementary Information 5 – Table S5: Summary of coded answers to open-ended questions**

Table S5. Summary of coded answers to the open-ended questions regarding lacking and positive support during treatment and during survivorship.

| **Domain** | **Topic** | **Description** | **Number of survivors mentioning lacking support areas** | | **Number of survivors mentioning positive support areas** | |
| --- | --- | --- | --- | --- | --- | --- |
|  |  |  | During treatment | During survivorship | During treatment | During survivorship |
| **Intrapersonal support** | Information on cancer / potential late effects | - Support in physical changes due to cancer, side effects of treatment | 6 | 3 |  |  |
|  |  | - Continuous information on new medical treatment options | 1 | 1 |  |  |
|  |  | - Support and information about menopause and cancer | 1 |  |  |  |
|  |  | - In-person communication of diagnoses | 1 |  |  |  |
|  |  | - Information about follow-up care, Follow-up care |  | 2 |  | 2 |
|  | Psychological support | - Psychological support; Access points for psychological assistance; mental support | 2 | 3 | 3 |  |
|  |  | - Self-help groups | 1 |  |  |  |
|  | Others | - Supporting and rehabilitating therapy | 2 | 3 |  |  |
|  |  | - Reduce the threshold for seeking therapy and support | 2 |  |  |  |
|  |  | - Nutrition counseling |  | 1 |  |  |
|  |  | - Assistance with leisure activities |  | 1 |  |  |
| **Interpersonal support** | Partnership support | - Help navigating romantic relationships |  | 1 | 1 |  |
|  | Family support | - Support in childcare | 3 |  | 1 | 1 |
|  | Family planning support | - Support and Information for those who desire to have children | 3 | 1 | 1 |  |
|  | Support for friends |  |  |  | 1 |  |
|  | Others | - Support and meetings with peers with cancer (e.g., same diagnosis, same gender) | 4 | 2 | 1 |  |
|  |  | - Support specifically for men and fathers |  | 1 |  |  |
| **Institutional / systemic support** | Educational support |  |  |  |  |  |
|  | Work-related support | - Support in a working situation |  | 1 |  |  |
|  | Insurance support | - Coverage of psychological therapy (sexual and couple therapy) | 1 |  |  |  |
|  |  | - 1:1 support from one qualified person | 1 |  |  |  |
|  |  | - More information, financial support | 1 | 1 |  |  |
|  |  | - Coverage of fertility treatments | 1 | 1 |  |  |
|  | Housing Support | - Support with household tasks | 2 |  |  |  |
|  | Others | - Assistance with bureaucracy (coordination of insurance, disability benefits, etc.) | 1 |  |  |  |
|  |  | - Food choices in the hospital | 1 |  |  |  |
|  |  | - Coordination among doctors and other medical staff | 1 | 1 |  |  |
|  |  | - Automated transmission of health reports |  | 1 |  |  |
|  |  | - Care in the hospital by doctors and medical staff; Support institutions (e.g., Krebsliga) |  |  | 4 | 1 |
